# Supplementary material for: Synthesis and characterization of attosecond light vortices in the extreme ultraviolet
Source: Nat Commun. 2016 Aug 30;7:12583. doi: 10.1038/ncomms12583 (PMC5013558; doi:10.1038/ncomms12583)
Supplement: Supplementary Information — Supplementary Figures 1-5, Supplementary Notes 1-4 and supplementary References. [file ncomms12583-s1.pdf]

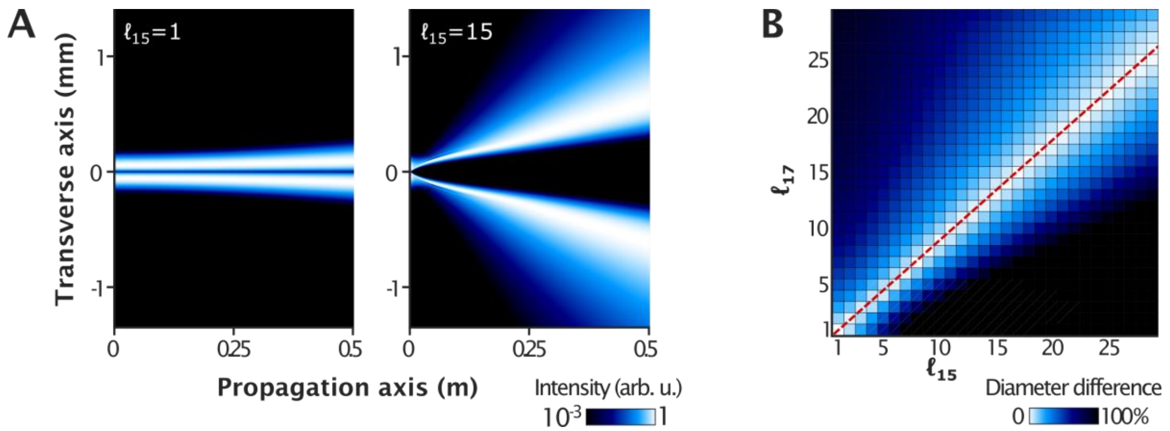

Supplementary Figure 1 | Numerical propagation of OAM beams. The waist of the harmonic field at focus is taken to be 60 μm. **(A)** Intensity of the 15-th harmonic during propagation along the optical axis. The field is propagated to the far-field from z = 0 to z = 0.5 m. The role of OAM in the field divergence is illustrated by taking either $\ell_{15} = 1$ (left) or $\ell_{15} = 15$ (right). The intensity is normalized to one for every value of z and is plotted in logarithmic scale.

(B) Relative difference between the diameters of H15 and H17 as a function of their respective OAM. The far-field profile of H15 and H17 are computed using Eq. (S2) and their diameter are gathered for every value of ℓ_{15} and ℓ_{17} between 1 and 30. For every couple of (ℓ_{15}, ℓ_{17}) values, the difference of their diameter is plotted in percentage of the diameter of H15. The dashed red curve is a linear fit of the combinations for which this difference is minimal, corresponding to rings of equal diameters. It corresponds to $\ell_{17} = 1.13\,\ell_{15} \simeq \frac{17}{15}\ell_{15}$.

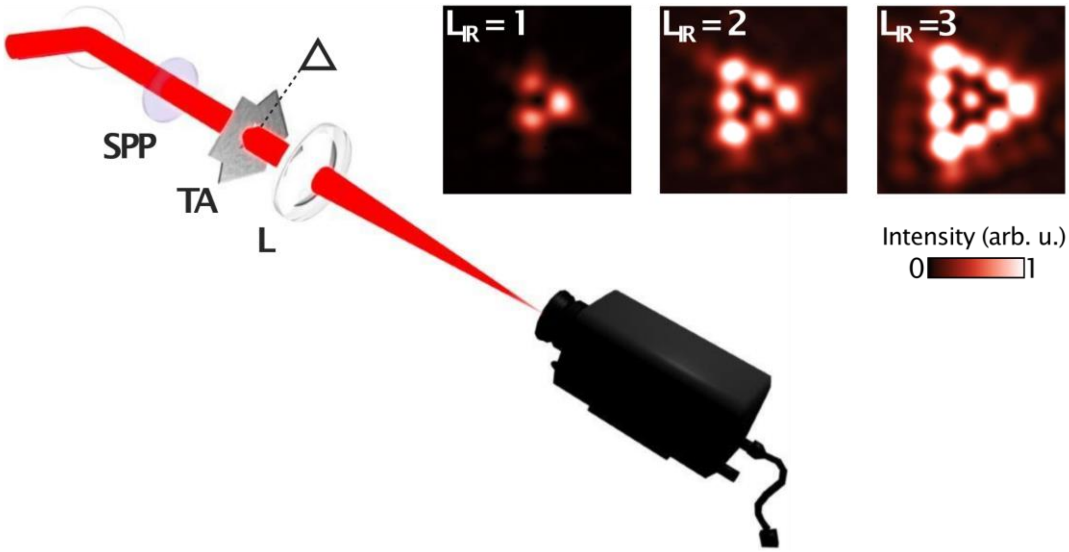

Supplementary Figure 2 | Measurement of the OAM of the driving laser. The collimated and spatial mode filtered femtosecond laser beam propagates through one or two of the spiral phase plates (SPP). A variable equilateral triangular slit is placed about 10 cm downstream. The diffracted beam is then focused by the same lens as for the HHG experiments. The focus is imaged on an Imagine Source CCD camera equipped with a x10 objective and a 160 mm long tube. The images recorded when placing the masks imposing a 2π, 4π and 6π azimuthal phases (corresponding respectively to $\ell_1 = 1$, $\ell_1 = 2$ and $\ell_1 = 3$) are displayed as insets. In the latter case, two masks imposing 2π and 4π are twinned to yield a 6π phase per round.

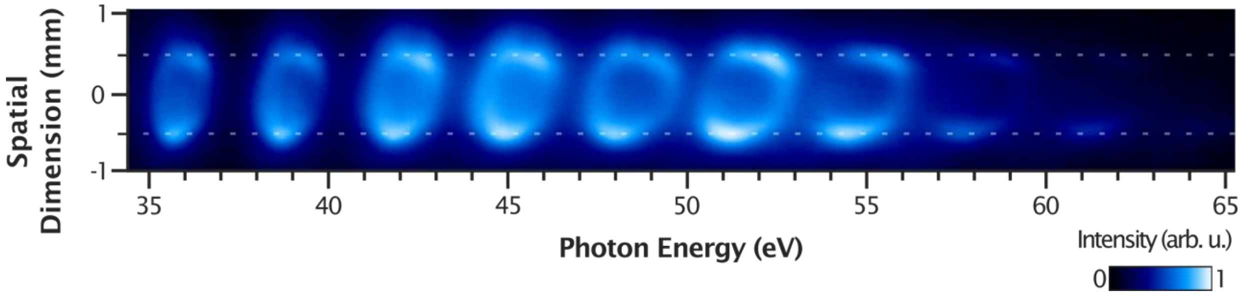

Supplementary Figure 3 | Experimental spectrum using neon as a generating gas. High harmonics are generated in a neon gas target using $\ell_1 = 1$ for the generating beam. The normalized intensity of harmonic orders 23rd to 41st is displayed. The intensity exhibits a clear ring profile, and we measure the average diameter to be 1.00 ± 0.05 mm, represented by the dashed white lines. This value is the same as what was measured in argon, which confirms our former observations on a broader spectral range. The OAM transfer law being verified, here the highest order carries an OAM of 41.

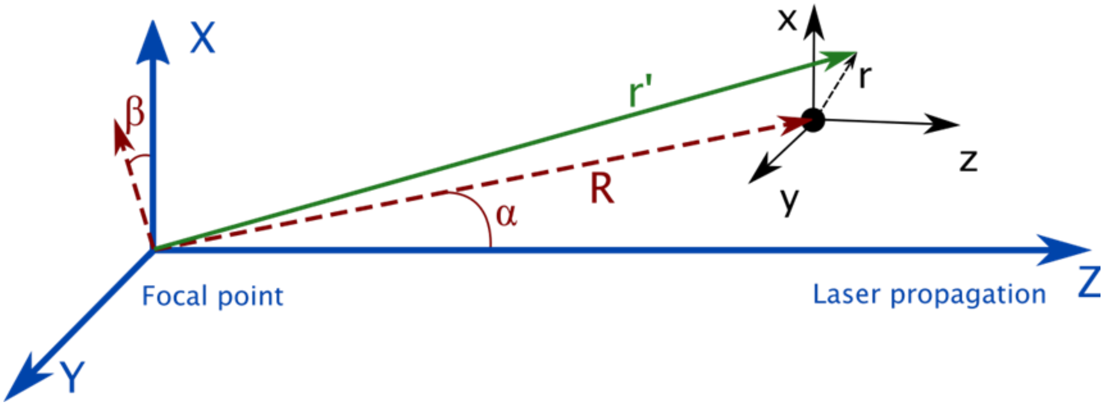

Supplementary Figure 4 | Geometry of the ionization of an atom by a twisted light beam. The coordinates *(X,Y,Z)* refer to the laboratory frame and *(x,y,z)* to the atomic frame centered on the atom nucleus.

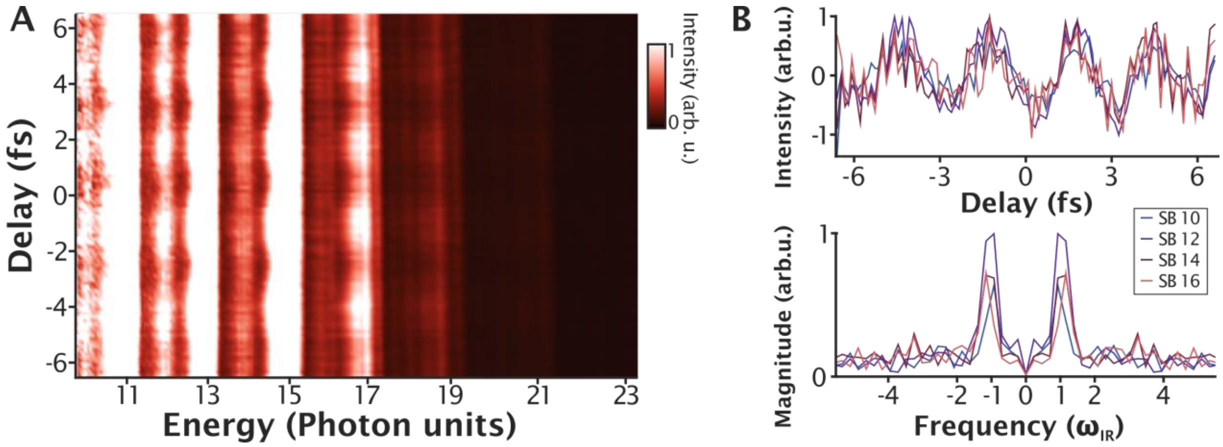

Supplementary Figure 5 | RABBIT trace when the dressing carries no OAM. (A) Two-color XUV+IR two-photon photoionization spectrogram of argon, in the same conditions as the Figure 3 of the main text, but when using no SPP in the dressing arm of the interferometer. The sidebands are still visible but now only present a single periodicity. **(B)** *(top)* Intensity of sidebands 10 to 16 as a function of the delay between the dressing and generation field. The DC component has been subtracted and the oscillations have normalized to 1. *(bottom)* Fourier transform of these oscillations in units of ω_{IR}, which is the angular frequency of the driving infrared field. The sidebands clearly do not show any $2\omega_{IR}$ component but oscillate at ω_{IR} (the IR period) due to the modulation of HHG by the dressing beam when it goes through the generating gas jet. This last effect is quite usual when using such a collinear scheme.

Supplementary Note 1 | Analytical expression of the ring diameter for an OAM beam

We focus on Laguerre-Gaussian (LG) modes, which are eigen solutions of the paraxial wave equation and form a natural set of helically phased light beams carrying all possible integer values of OAM[2]. We restrict ourselves to LG modes with a radial index p = 0, thus showing a single intensity ring. We further make the hypothesis that HHG driven by such an IR LG beam with OAM ℓ_1 leads to a comb of XUV LG modes, with the q-th order carrying ℓ_q units of OAM. Using cylindrical coordinates (R,θ,z), where R is the distance from the optical axis, z the distance along the optical axis from the focal point and $z_R = \pi w_0^2/\lambda_1$ the Rayleigh range, w_0 the beam waist and λ_1 the IR wavelength, the spatial profile of the electric field of a LG mode reads, up to normalization constants :

$$E_\ell(R,\theta,z) = \frac{e^{i\ell\theta}}{w(\lambda,z)}\left(\frac{R}{w(\lambda,z)}\right)^{|\ell|} e^{\left(-i\frac{\pi R^2 z}{\lambda_1\left(z_R{}^2+z^2\right)}-\frac{R^2}{w(\lambda,z)^2}-i\psi^L_{\ell,0}\right)}, \tag{1}$$

where ℓ corresponds to the OAM per photon in this mode, $w(\lambda,z) = w_0\sqrt{1+(z/z_R)^2}$ is the equivalent of the waist for a Gaussian beam, and $\psi^L_{\ell,0} = \left(|\ell|+1\right)\tan^{-1}(z/z_R)$ is the equivalent of the Gouy phase. Let us consider HHG driven by such an infrared LG beam and assume that the q-th order is also a LG mode with an OAM ℓ_q. The corresponding intensity writes

$$I_{\ell_q}(R,\theta,z) = \frac{1}{w^2(\lambda,z)}\left(\frac{R}{w(z)}\right)^{2|\ell_q|} e^{\left(-\frac{2R^2}{w^2(\lambda,z)}\right)} \tag{2}$$

The maximum intensity along the radius is obtained at $\partial I_{\ell_q}/\partial R = 0$, i.e.

$$\left(\frac{2|\ell_q|}{R}-\frac{4R}{w^2(z)}\right)\left(\frac{R}{w(z)}\right)^{2|\ell_q|} e^{\left(-\frac{2R^2}{w^2(z)}\right)} = 0 \tag{3}$$

The solution to this equation is:

$$R_{max} = w(\lambda,z)\sqrt{\frac{|\ell_q|}{2}} \tag{4}$$

For each order, the maximum intensity is located at:

$$R_{max}(\lambda_q,z) = w(\lambda_q,z)\sqrt{\left|\frac{\ell_q}{2}\right|} = w(\lambda_q,0)\sqrt{1+\left(\frac{z\lambda_q}{\pi w(\lambda_q,0)^2}\right)^2}\sqrt{\left|\frac{\ell_q}{2}\right|} \tag{5}$$

where $\lambda_q = \lambda_1/q$ is the wavelength of the q-th order. Without getting into details about the generation process, it can be assumed that the intensity profile of the XUV beam is maximal where the intensity of the IR field is maximal as well. This is predicted in the Strong Field Approximation (SFA)[3] or directly by solving the Time Dependent Schrödinger Equation (TDSE)[4]. Equating the position of the maxima of the IR and the XUV yields

$w(\lambda_q,0)\sqrt{\frac{\ell_q}{2}} = w(\lambda_1,0)\sqrt{\frac{\ell_1}{2}}$. Taking into account $\lambda_q = \lambda_1 / q$, one finally gets the expression for the radius of the rings:

$$R_{max}(q,z) = w(\lambda_1,0)\sqrt{\left|\frac{\ell_1}{2}\right|}.\sqrt{1+\left(\frac{z\lambda_1}{\pi w(\lambda_1,0)^2}\frac{\ell_q}{q\ell_1}\right)^2}. \quad (6)$$

We retrieve the fact that the OAM of the beam rules its divergence. In particular, the multiplicative law $\ell_q = q\times\ell_1$ yields a constant ring diameter over the whole spectrum while $\ell_q = \ell_1$ ($\ell_1 \neq 0$) would give a diameter decreasing with q.

When driving HHG with regular Gaussian beams, the central part of the XUV beam shows an increasing divergence with q. This regular behavior is mainly a consequence of the HHG process itself combined to the λ-dependence of light propagation to the far field. Here we show that the helical phase modifies this regular behavior. Inspecting the divergence of harmonics thus answers the controversy about the transfer law of OAM, without resorting to any diffractive element or interference scheme.

Supplementary Note 2 | Numerical propagation of OAM beams

In this part we illustrate the link between the evolution of the ring diameter in the far-field with the harmonic order, and the OAM carried by the field. We first take the 15th harmonic as an example. We define its intensity at focus as the standard ring-shaped distribution of an LG mode. Then we define its spatial phase as $\ell_{15}\theta$ and study the effect of the value of ℓ_{15} on its propagation (Supplementary Fig. 1A). We observe very different diameters in the far field, depending on the ℓ value.

Now, the same computation is done systematically for several values of ℓ_{15} between 1 and 30. For each one, we gather the diameter of H15 in the far-field (z = 0.5 m). Doing the same for H17 allows us to compare the diameters of the two harmonics for each couple of (ℓ_{15},ℓ_{17}) values, as shown in Supplementary Fig. 1B. We see that the diameters are the closest along a line whose slope is $\ell_{17} = 1.13\ell_{15} \simeq \frac{17}{15}\ell_{15}$. We repeated these calculations for harmonics orders q between 13 and 25 and obtained the general result that the OAM transfer law should obey the rule $\ell_{q+2} = \frac{q+2}{q}\ell_q$ for harmonics to show constant diameters throughout the spectrum.

Supplementary Note 3 | Direct measurement of the OAM of the driving laser at the focal spot

The measurement of the OAM in the visible domain usually relies on interferometric or diffractive schemes[1]. We here chose to check the behavior of our phase masks placing a triangular slit in the path of the beam. The experimental scheme used for HHG is just slightly modified: a triangular slit is placed before the focusing lens and the beam at focus is imaged on a CCD camera equipped with a microscope objective. As expected from the theory, when choosing a size of the variable slit comparable to the waist of the laser, we obtained a series of spots along a triangular pattern (see Supplementary Fig. 2), whose number is related to the OAM carried by the beam. This observation was only made when the incoming beam presented a very close to flat wavefront. For instance, constraining a mirror in its mount ruins this structure. This online diagnosis secured the quality of the incoming beam for HHG.

Supplementary Note 4 | RABBIT with beams carrying OAM

Here we propose to analyze how the OAM of a twisted photon beam transfers to electrons through photoemission of atoms, with an emphasis on the preserved validity of the dipolar selection rule. The formalism and notations below are closely related to the ones used in Ref.[5].

We first define the vectors which represent the electron and nucleus positions in the laboratory and the atomic frames, respectively. In the laboratory frame, the nucleus is located at $\mathbf{R}=(\mathrm{R},\alpha,\beta)$ and the electron at $\mathbf{r'}=(\mathrm{r'},\theta',\phi')$, while in the atomic frame the nucleus is at (0, 0, 0) and the electron at $\mathbf{r}=(\mathrm{r},\theta,\phi)$. Bold letters denote vectors. The two sets of coordinates are related by $\mathbf{r'}=\mathbf{R}+\mathbf{r}$, as shown in the Supplementary Fig. 4.

Rewriting Eq. (2) of Ref.[5] for p = 0 and using the vectors defined above, we obtain for the expression of the vector potential at the electron position $\mathbf{r'}$:

$$\mathbf{A}(\mathbf{r'},t)\propto\frac{1}{w(z')}\left(\frac{\rho'}{w(z')}\right)^{|\ell_1|}\exp\left(-\frac{\rho'^2}{w^2(z')}\right)\exp\left[i\ell_1\phi'-i\frac{\rho^2 z'}{w^2(z')z_R}-i\psi^{L}_{\ell,0}(z')\right]. \quad (7)$$

Here, we defined $\rho'=$ r'sinθ', $z'=$ r'cosθ', and the other quantities are the same as in Eq. (1).

The photoelectron angular distribution for the absorption of one photon is given by the differential cross section which, according to the Fermi Golden rule, requires computing such a matrix element:

$$\int_{\mathbf{r}}\psi_f^*(\mathbf{r})\left[\vec{A}(\mathbf{r'},t).\mathbf{p}\right]\psi_i(\mathbf{r})\,d^3\mathbf{r}, \quad (8)$$

where $\psi_i(\mathbf{r})$, $\psi_f(\mathbf{r})$ are the initial and final state wavefunctions, respectively.

Two limiting cases appear:

(i) $R\sin\alpha = 0$;
(ii) $R\sin\alpha >> r$ (≈ 1 a.u).

In the first one, we reach the conditions of Ref.[5] with $\phi' = \phi$, which means that the angles appearing in Eq. (7) are the same as the ones in the integral of Eq. (8). Therefore, one obtains nondipolar selection rules (Eq. (6) of [5] which depend on the value ℓ_1 of the OAM, reminiscent of the marked variations of A at the atomic scale, in the vicinity of the beam axis. In the other case, we can assume $\mathbf{r'} \approx \mathbf{R}$, which validates the dipole approximation. There, $\mathbf{A}(\mathbf{r'}, t)$ at the atomic scale becomes independent of the electron's coordinates and just acts as a position-dependent amplitude and phase factor, $\xi(\mathbf{R})$, on the usual dipole matrix element. It leads to the standard $\Delta L = \pm 1$ rule, which is notably assumed in various experiments involving single twisted photons transferring their OAM to an ensemble of atoms[1].

This implies that, apart from a tight region in space close to the laser propagation axis, where the electric field is very small, twisted photons act like "normal" (i.e. plane-wave) ones, except that they carry a $\mathbf{R}$-dependent phase, which adds up in multiphoton processes like HHG or RABBIT. Thus, the q-th harmonic order having an OAM of $\ell_q = q\times\ell_1$, the $\mathbf{R}$-dependent RABBIT oscillations of the sideband will be modulated as

$$SB_{q+1}(\omega,\mathbf{R}) = \cos\left[2\omega\tau_0 + \varphi_{q+2} - \varphi_q + \left(\ell_{q+2} - \ell_q - 2\ell_d\right)\, \xi(\mathbf{R})\right], \tag{9}$$

where ℓ_d is the OAM of the dressing field and φ_{q+2}, φ_q the harmonic spectral phase. If the dressing beam is homogeneous and shows almost flat phase fronts ($\ell_d = 0$ for instance) the last phase term covers 0-2π and washes out the oscillations when averaging over the positions of the atom, as a temporal jitter would do. On the contrary, dressing with an IR carrying an OAM $\ell_d = \frac{\ell_{q+2} - \ell_q}{2}$ ensures keeping the oscillatory behavior of the sidebands.
We illustrate this point by measuring a RABBIT trace where $\ell_q = q$ and $\ell_d = 0$, i.e. by dressing the vortex harmonics with an IR carrying no OAM. This is done experimentally by putting the SPP only in one arm of the Mach-Zender interferometer. The results shown in Supplementary Fig. 5 show no oscillation, as expected.

Supplementary references

1. Yao, A. M. & Padgett, M. J. Orbital angular momentum: origins, behavior and applications. *Adv. Opt. Photon.* **3**, 161–204 (2011).
2. Allen, L., Beijersbergen, M. W., Spreeuw, R. J. C. & Woerdman, J. P. Orbital angular momentum of light and the transformation of laguerre-gaussian laser modes. *Phys. Rev. A* **45**, 8185–8189 (1992).
3. Lewenstein, M., Balcou, P., Ivanov, M., L'Huillier, A. & Corkum, P. B. Theory of high-order harmonic generation by low-frequency laser fields. *Phys. Rev. A* **49**, 2117 (1994).
4. Kulander, K. C., Schafer, K. J. & Krause, J. L. Time-Dependent Studies of Multiphoton Processes. In Gavrila, M. (ed.) *Atoms in Intense Laser Fields*, 247 (1992).
5. Picón, A. *et al.* Photoionization with orbital angular momentum beams. *Opt. Express* **18**, 3660–3671 (2010).
